# Integrative Use of Cannabidiol, Melatonin, and Oxygen–Ozone Therapy in Triple-Negative Breast Cancer with Lung and Mediastinal Metastases. A Case Report

**DOI:** 10.3390/reports9010028

**Published:** 2026-01-19

**Authors:** Cristina Aguzzi, Paola Zuccoli, Alessandro Fanelli, Alessandra Mammone, Massimo Nabissi, Margherita Luongo

**Affiliations:** 1 School of Pharmacy, University of Camerino, Camerino, 62032 Macerata, Italy; cristina.aguzzi@unicam.it (C.A.); massimo.nabissi@unicam.it (M.N.); 2 Department of Radiotherapy, Institute Ecomedica Empoli, Empoli, 50053 Firenze, Italy; paola.zuccoli@ecomedica.it (P.Z.);; 3 Specialization School of Hospital Pharmacy, University of Perugia, 06123 Perugia, Italy; 4 “Maria Guarino” Foundation-AMOR No Profit Association, 80078 Pozzuoli, Italy; 5 Scuola di Medicina e Chirurgia, Università degli Studi della Campania Luigi Vanvitelli, 81100 Naples, Italy

**Keywords:** cancer metastasis, case report, cannabidiol, melatonin, oxygen-ozone, breast cancer, integrative therapy

## Abstract

**Background and Clinical Significance**: Breast cancer is the most frequent malignancy in women. Metastatic breast cancer is considered a treatable but incurable condition, with a median overall survival of only 2–3 years. Among its subtypes, triple-negative breast cancer (TNBC) accounts for a high proportion of breast cancer-related deaths. It is characterized by an aggressive clinical course, early recurrence, and a strong propensity for visceral and brain metastases. **Case Presentation**: We report the case of a Caucasian woman who developed systemic disease recurrence with lung and mediastinal lymph node metastases, occurring two years after her primary diagnosis and treatment for TNBC. The patient received three months of chemotherapy combined with an adjuvant integrative protocol consisting of melatonin, cannabidiol, and oxygen–ozone therapy. This combined approach led to the complete disappearance of the lung nodules. Subsequently, stereotactic radiotherapy was performed and, in association with the ongoing integrative treatment, resulted in a significant reduction in mediastinal adenopathy. Introduction of immunotherapy, supported continuously by the same adjuvant strategy, achieved a complete and durable remission. Strikingly, the patient remained disease-free five years after the diagnosis of lung and mediastinal metastases. **Conclusions**: This clinical case highlights the potential benefit of using melatonin, cannabidiol, and oxygen–ozone therapy as part of an integrative approach in patients with aggressive metastatic TNBC. While it is not possible to establish causality from a single case, the sustained remission observed suggests that such unconventional adjuvant strategies could play a supportive role in enhancing the efficacy of standard oncologic therapies.

## 1. Introduction and Clinical Significance

Breast cancer is the most common malignancy in women and accounts for the 30% of female cancers worldwide [1,2]. Despite a 5-year survival rate of 90%, breast cancer remains the leading cause of cancer death in women aged 20 to 59 years old [1]. A family history of cancer and genetic predisposition—particularly high-penetrance mutations in *BRCA1* and *BRCA2*, as well as other genes like *CDH1*, *TP53*, and *PTEN*—significantly increase individual risk; however, other risk factors exist. For example, certain chemotherapy regimens or exposure to chest radiation are an established risk factor [3,4]. Other well-documented contributors include reproductive history, hormonal therapy, and lifestyle factors such as obesity, physical inactivity, alcohol consumption, a low-fiber diet, and smoking [3]. The clinical classification of breast cancers is traditionally based on the expression of estrogen receptors (ERs), progesterone receptors (PRs), and human epithelial growth factor receptor 2 (HER2). However, these markers are now used to define the intrinsic molecular subtypes, which provide deeper insights into tumor biology and prognosis: Luminal A (ER/PR positive, HER2 negative, low Ki-67), Luminal B (ER/PR positive, with either HER2 positivity or high Ki-67), HER2-enriched, and Basal-like [5]. The latter largely overlaps with triple-negative breast cancer (TNBC), which lacks ER, PR, and HER2 expression and typically exhibits a more aggressive clinical course [6]. Early breast cancer is cancer that is contained in the breast or that has only spread to the axillary lymph nodes. TNBCs represent about 10% of all breast cancers but account for a disproportionally high number of breast cancer-related deaths due to their aggressive nature, early recurrence, and high propensity for lung and brain metastases [2,7]. In patients with germline *BRCA1/2*-mutated TNBC, PARP inhibitors (e.g., Olaparib, Talazoparib) have shown significant benefit when used following or in addition to standard systemic therapies, rather than in isolation. In the metastatic setting, the OlympiAD and EMBRACA trials demonstrated efficacy in patients previously treated with anthracyclines and taxanes. More recently, different trials confirmed that adjuvant Olaparib improves survival in high-risk patients who have completed standard neoadjuvant or adjuvant chemotherapy [8,9,10]. Recent evidence also points to the potential of unconventional adjuvant therapies. Oxygen–ozone (O_2_O_3_) has been shown to inhibit cancer cell growth and induce cancer cell death in vitro, with no effect on normal cells. In vivo, ozonated water induced tumor necrosis in a rectal cancer mouse model, and intratumoral oxygen–ozone injections prolonged survival in patients with recurrent glioblastoma [11]. The antitumor effect of ozone appears linked to the induction of oxidative stress through reactive oxygen species (ROS), which cancer cells, already under oxidative pressure, are less able to counteract compared with normal cells [12]. Cannabidiol (CBD), a non-psychomimetic phytocannabinoid extracted by *Cannabis sativa* plants, has also shown anticancer properties in preclinical models in addition to palliative effects such as analgesic, antiemetic, and antidepressant actions [13]. Nabiximols, a pharmaceutical preparation enriched in CBD and Δ^9^-tetrahydrocannabinol, displayed anticancer effects in a clinical trial [14]. Specifically, in breast cancer models, CBD reduced cell viability, proliferation, migration, and invasiveness while enhancing the efficacy of chemotherapeutic drugs [15,16]. Melatonin (MLT) has similarly demonstrated anticancer properties, both in vitro and in vivo, by inducing apoptosis, inhibiting proliferation and metastasis, potentiating conventional therapies, and reducing their adverse effects [17]. Moreover, the combination of CBD, MLT, and O_2_/O_3_ therapy has been shown to negatively impact pancreatic ductal adenocarcinoma in both in vitro and in vivo models [18]. Here, we report the case of a woman with TNBC who developed lung and mediastinal lymph node metastases and received standard chemo-, radio-, and immunotherapy in combination with CBD, MLT, and O_2_/O_3_ therapy as adjuvant treatment.

## 2. Case Presentation

The patient was a Caucasian woman with a positive oncological family history, as both her paternal grandmother and paternal uncle had developed breast cancer. In 2017, four years after her third pregnancy, at the age of 42, she reported right arm pain and detected a breast nodule on self-examination. A mammogram confirmed the presence of a lesion, and subsequent diagnostic workup led to the diagnosis of triple-negative breast cancer with a Ki-67 proliferation index of 80%. In April 2017, she underwent a right quadrantectomy for an invasive ductal carcinoma, grade 3 (CDI G3), staged as pT1c pN0 M0. Adjuvant treatment included chemotherapy with four cycles of EC (Epirubicin and Cyclophosphamide), followed by 12 weekly cycles of Paclitaxel, and radiotherapy with a total dose of 50 Gy delivered in 25 fractions of 2 Gy each. A genetic analysis revealed a pathogenic *BRCA1* mutation (exon 20, c.5266dupC, p.Gln1756Profs*74). Consequently, in April 2018, the patient underwent prophylactic bilateral adnexectomy.

**Figure 1:**
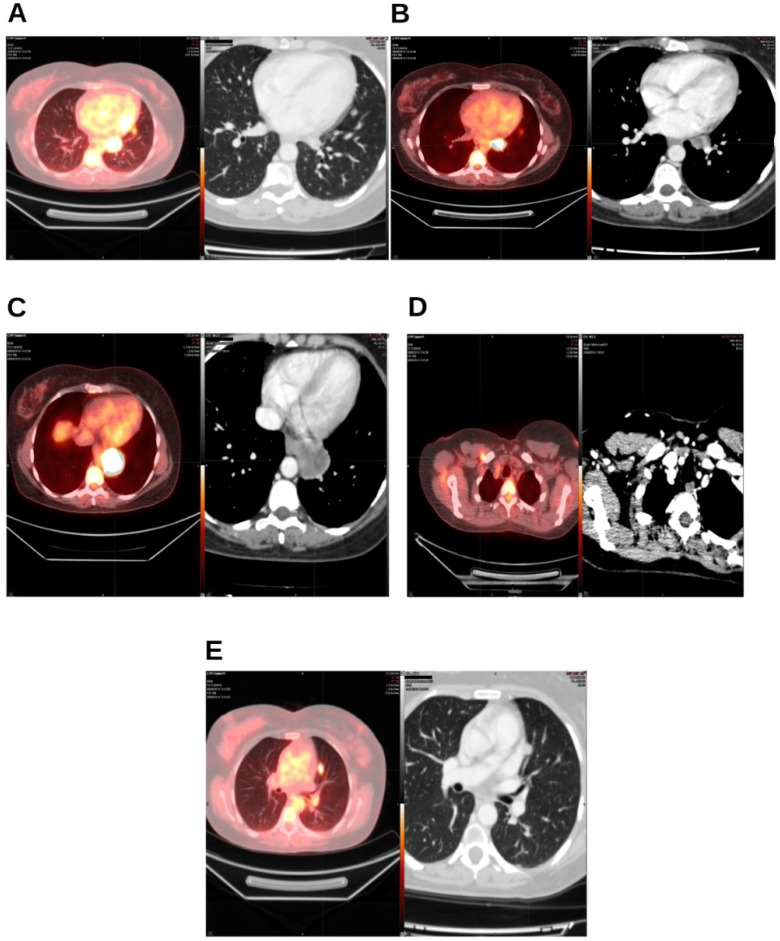
Diagnostic tests before the start of radiotherapy treatment. On the left, the PET-TC from August 2019; on the right, the CT from August 2019. (**A**) Lingular segment; (**B**) left hilar lymph node; (**C**) left paraesophageal nodule; (**D**) right retropectoral lymph node; (**E**) nodule in left parascissural segment.

In August 2019, two years after the initial diagnosis of triple-negative breast cancer and subsequent surgery, the patient was found to have lung and mediastinal lymph node metastases. The diagnosis was established through total-body positron emission tomography (PET) performed in June 2019 and later confirmed by computerized axial tomography (CT scan) in June 2019 (Figure 1A–E).

In September 2019, the patient initiated chemotherapy with carboplatin and gemcitabine administered every 21 days. However, treatment was discontinued in November 2019 due to adverse side effects.

During the same period (September 2019), the patient also began an integrative protocol consisting of MLT, CBD, and O_2_/O_3_ therapy. The O_2_/O_3_ therapy was administered via rectal insufflation of an oxygen–ozone mixture (97% oxygen, 3% ozone) at a concentration of 80 µg/mL, with a volume of 2.5 mL/kg, four times per week for three months, followed by a three-month break. MLT was initially taken as 100 mg/day, with the dose increased by 100 mg every three days up to 2 g/day (500 mg tablets, 4 per day). CBD was administered at 200 mg/day during the three months of O_2_/O_3_ therapy and increased to 400 mg/day during the three-month breaks. This integrative regimen with MLT, CBD, and O_2_/O_3_ is still ongoing, although O_2_/O_3_ therapy was reduced to two sessions per week after the patient achieved complete remission.

In October, left para-aortic and hilar lymph nodes were treated with stereotactic radiotherapy. At that time, the lung nodules were no longer detectable. For the treatment, the patient underwent a contrast-enhanced CT simulation acquired in “Breath hold” using Active Breathing Coordinator (ABC) System with a slice thickness of 1 mm. The CTV (Clinical Target Volume) and OAR (organ-at-risk) delineation was performed on a fused imaging between venous-phase CT simulation and diagnostic PET imaging. A 2 mm margin was applied to each target to create the PTV (Planning Target Volume). The VMAT (Volumetric Modulated Arc Therapy) treatment was delivered by 6 MV Linac. CBCT (Cone Beam Computed Tomography) was employed to control patient set-up before each fraction. Stereotactic radiotherapy was delivered with a total dose of 30 Gy at the 80% isodose (isocenter dose 37.5 Gy) in three fractions using a monoisocentric technique.

**Figure 2:**
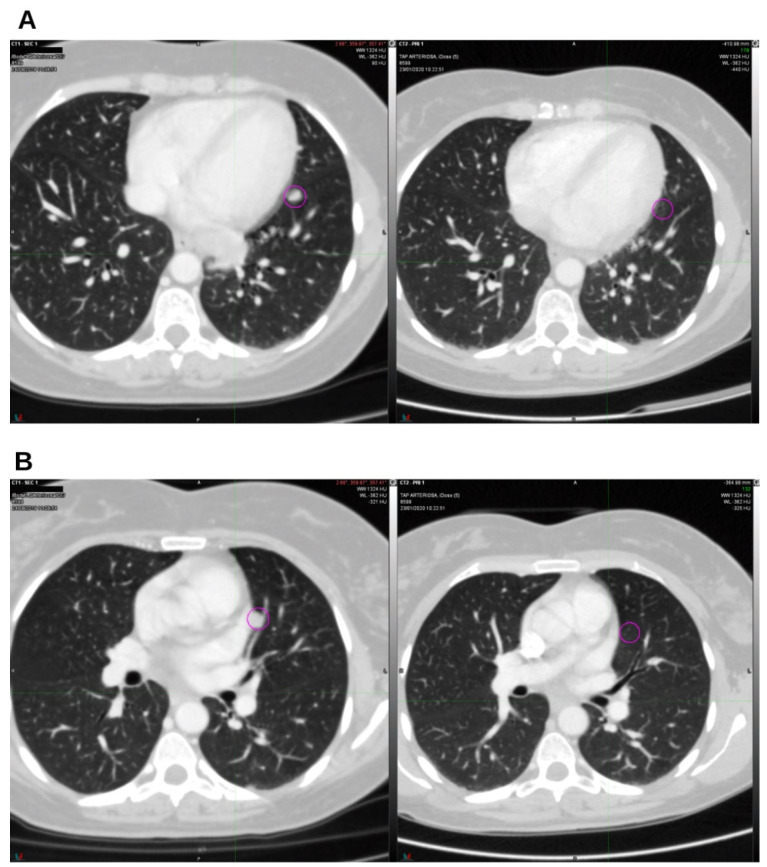
On the left, CT from August 2019; on the right, CT from January 2020. The lingular (**A**) and parascissural (**B**) nodules were in complete regression. Nodes in the circles.

**Figure 3:**
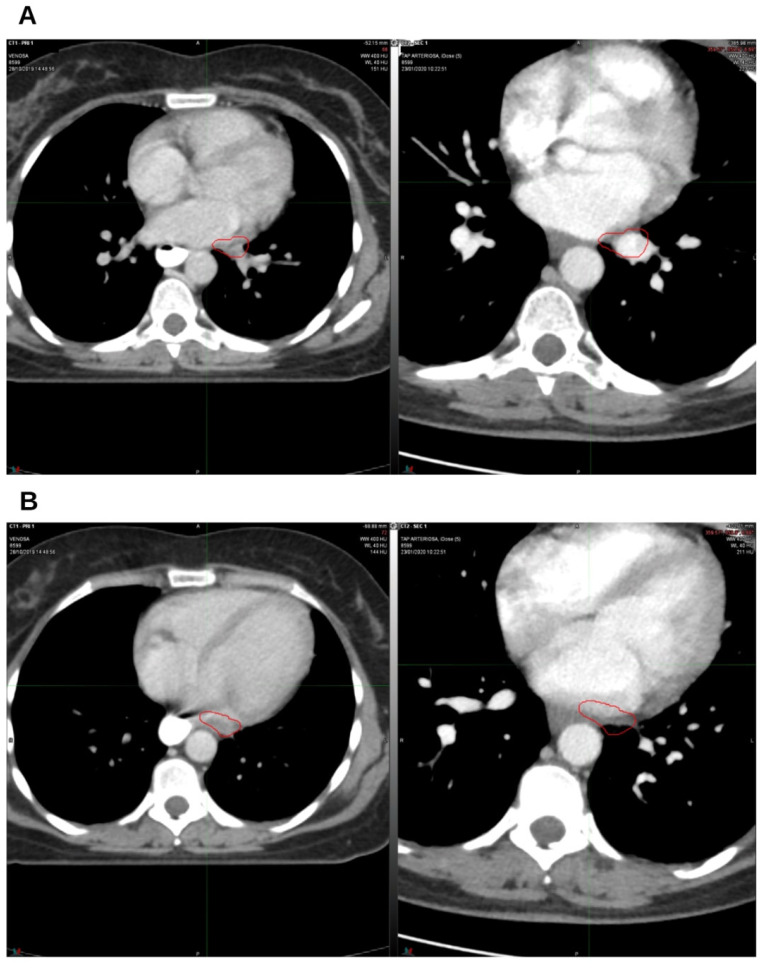
The first follow-up at 3 months off. On the left, CT from October 2019, pre-radiation therapy treatment; on the right, CT from January 2020. (**A**) Hilar lymph node; (**B**) left para-aortic lymph node. The indicated region highlights the boundary of the treated target.

On January 2020, a CT scan confirmed the absence of the two lung nodules (Figure 2A,B) and demonstrated a reduction in lymph node volume compared to October 2019 (Figure 3A,B).

In March 2020, the patient also started Olaparib, a PARP inhibitor, at a dosage of 600 mg/day (150 mg tablets, 4/day). Due to persistent nausea, the dose was progressively reduced to 300 mg/day and then to 150 mg/day during February and March 2023. In June 2023, treatment was completely discontinued because of leukopenia. Although her physician recommended resuming therapy, the patient declined until March 2024, when she agreed to restart Olaparib.

In addition, beginning in July 2020, the patient was supplemented with vitamin D at a dosage of 50,000 IU once weekly.

**Figure 4:**
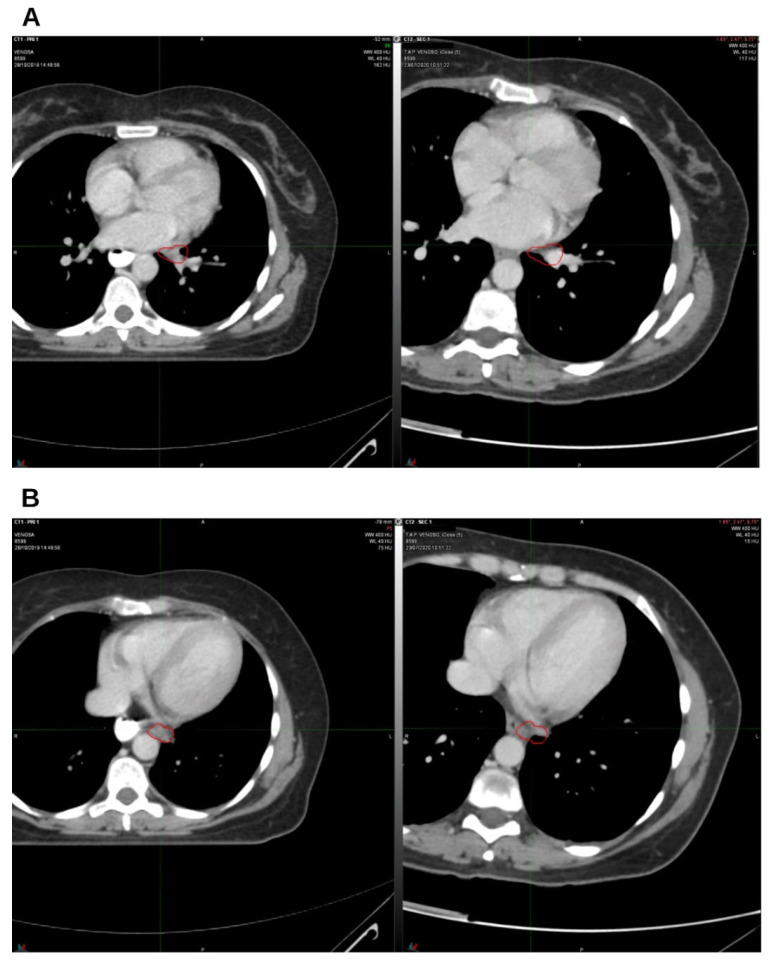
The third follow-up at 9 months off. On the left, CT from October 2019, pre-radiation therapy treatment; on the right, CT from July 2020. (**A**) Hilar lymph node; (**B**) left para-aortic lymph node. The indicated region highlights the boundary of the treated target.

A CT scan performed in April 2020 demonstrated a further reduction in the size of the left para-aortic and hilar lymph nodes. An additional decrease was documented in a subsequent CT scan in July 2020 (Figure 4A,B).

**Figure 5:**
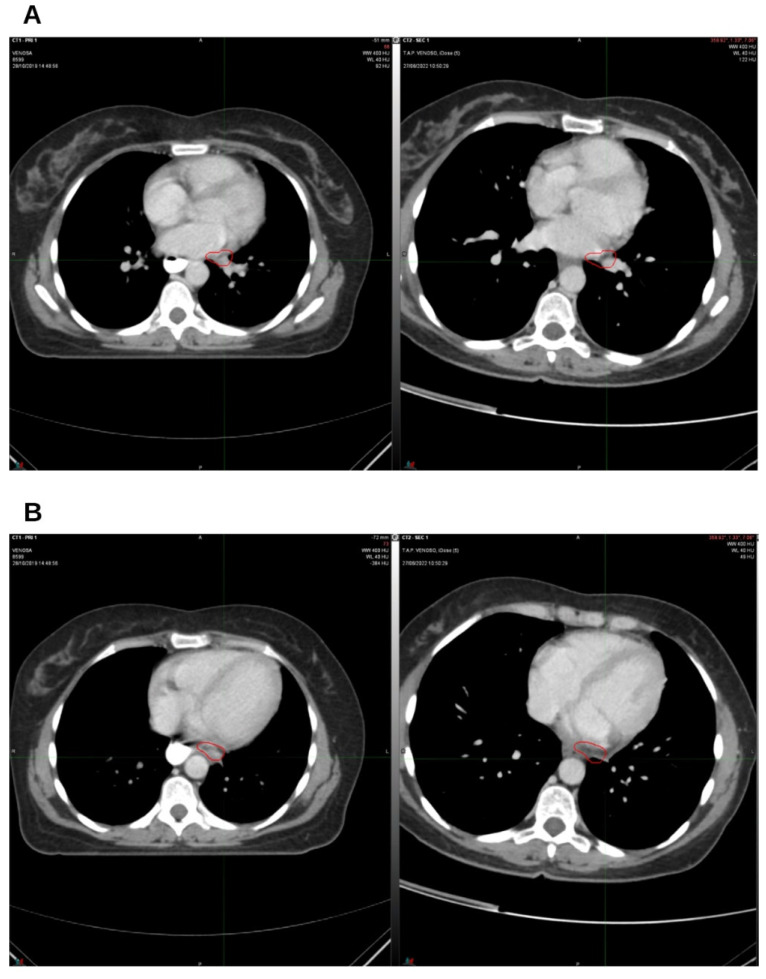
On the left, CT from October 2019, pre-radiation therapy treatment; on the right, CT from August 2021. (**A**) Hilar lymph node; (**B**) left para-aortic lymph node. The indicated region highlights the boundary of the treated target.

**Figure 6:**
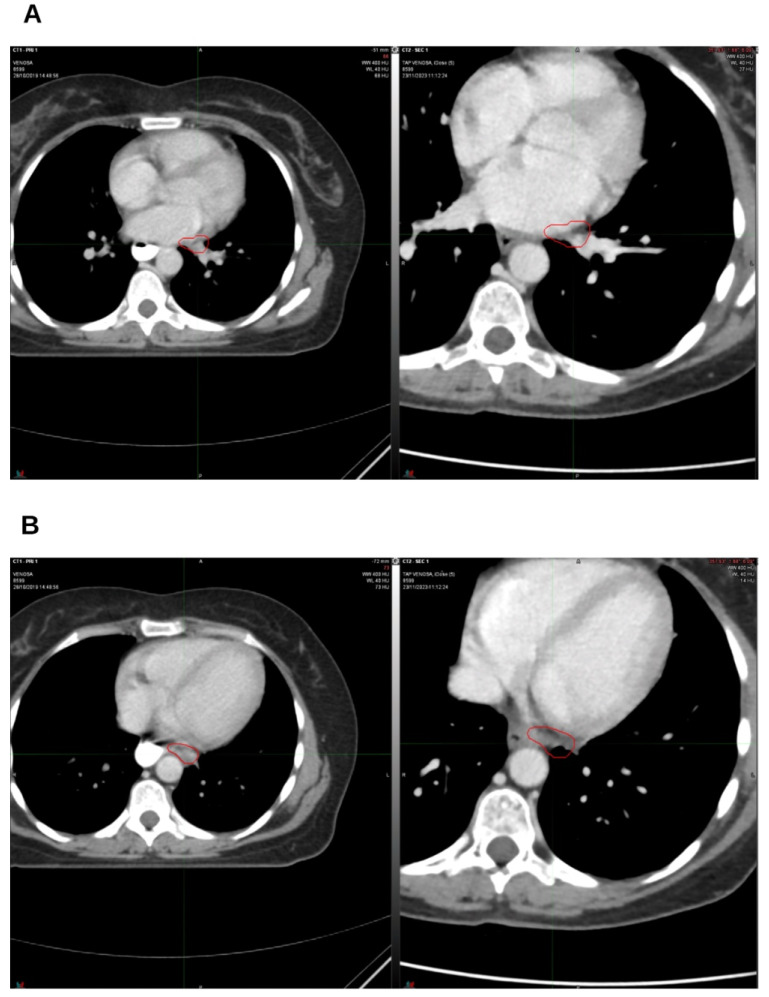
On the left, CT from October 2019, pre-radiation therapy treatment; on the right, CT from June 2022. (**A**) Hilar lymph node; (**B**) left para-aortic lymph node. The indicated region highlights the boundary of the treated target.

**Figure 7:**
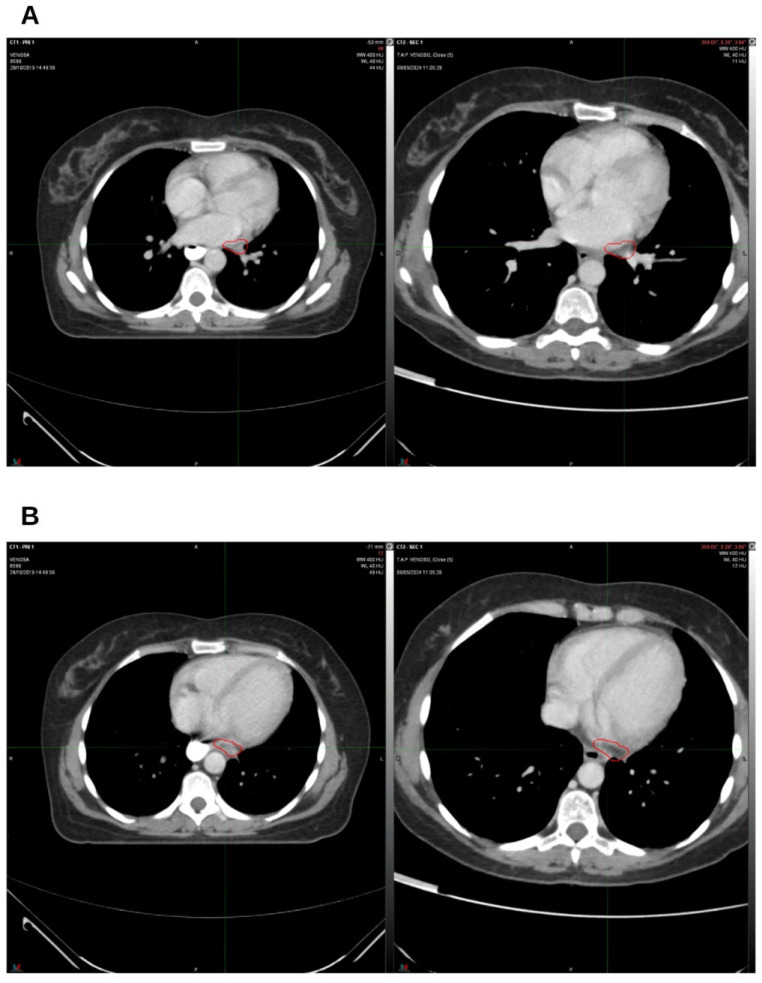
On the left, CT from October 2019, pre-radiation therapy treatment; on the right, CT from November 2023. (**A**) Hilar lymph node; (**B**) left para-aortic lymph node. The indicated region highlights the boundary of the treated target.

**Figure 8:**
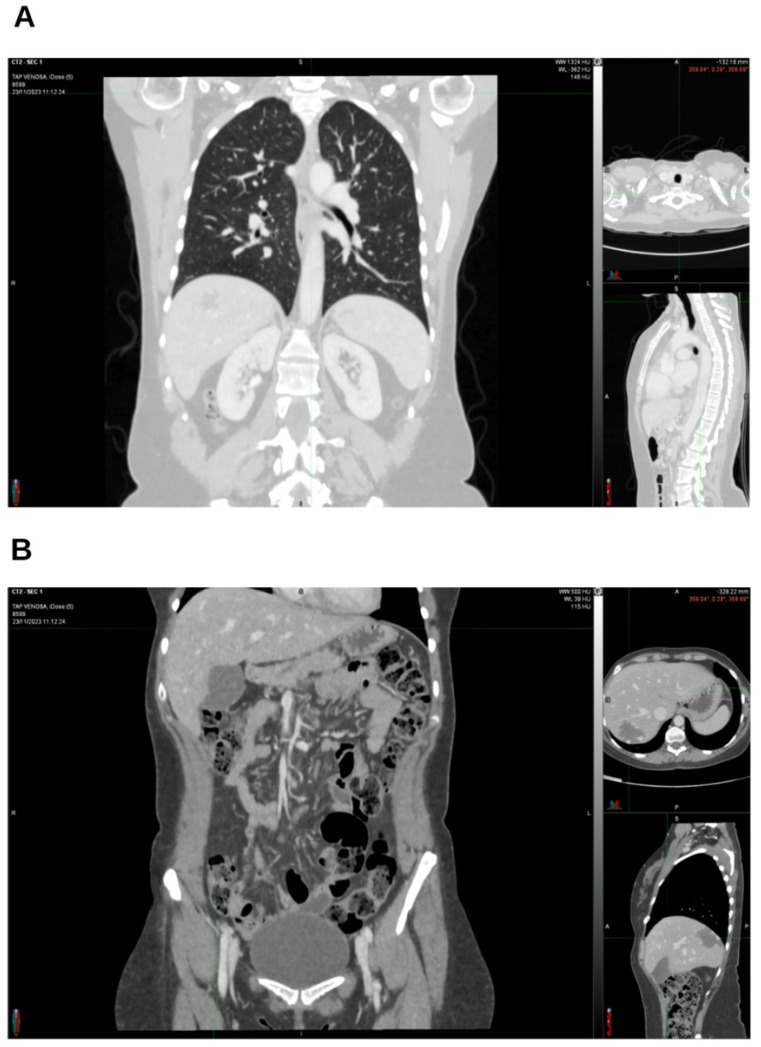
In November 2023, the lung (**A**), abdomen, and pelvis (**B**) CT scan was still negative.

In April 2021, the total-body CT scan showed a complete response, with no detectable disease (Figure 5A,B). Subsequent follow-up analysis performed in 2022 (Figure 6A,B) and 2023 (Figure 7A,B) confirmed the absence of recurrence and of radiological signs of disease relapse (Figure 8A,B).

**Figure 9:**
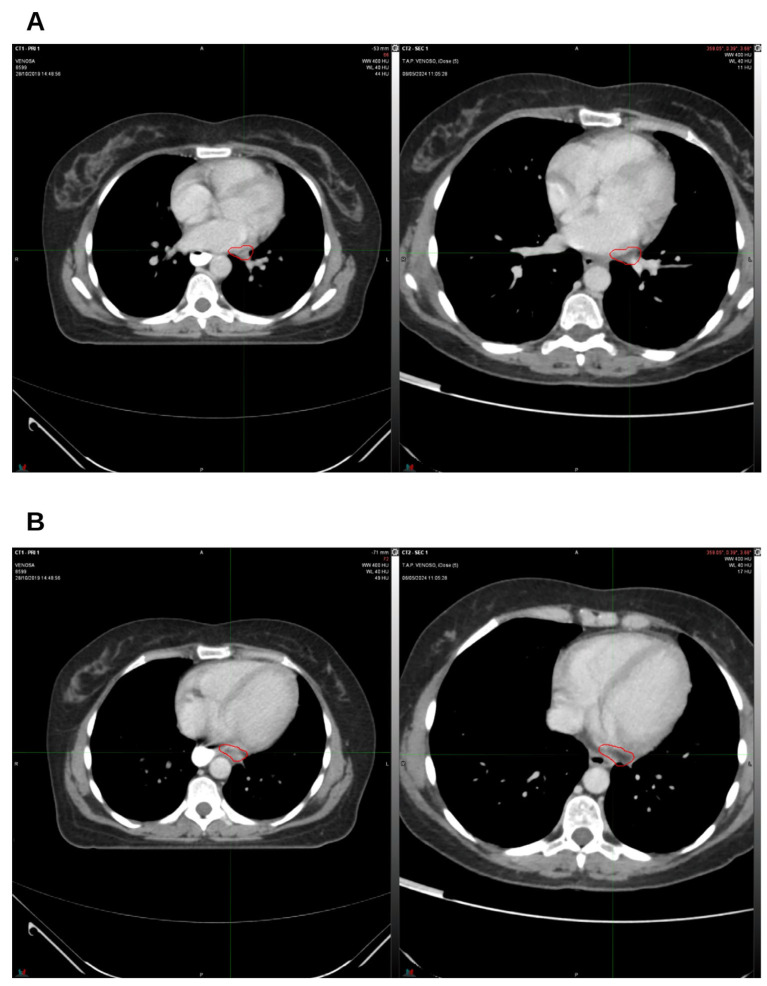
The last follow-up. On the left, CT from October 2019, pre-radiation therapy treatment; on the right, CT from May 2024. (**A**) Hilar lymph node; (**B**) left para-aortic lymph node. The indicated region highlights the boundary of the treated target.

In 2024, the patient continued treatment with Olaparib 600 mg/day, in combination with integrative therapy consisting of O_2_/O_3_ therapy, MLT (2 g/day), and CBD (200 mg/day during the 3 months of O_2_/O_3_ therapy, increased to 400 mg/day during the 3-month breaks). The most recent CT scan performed in May 2024 (Figure 9A,B) confirmed the persistence of a complete response, with no evidence of disease recurrence, five years after the diagnosis of lung and mediastinal metastases.

## 3. Discussion

Herein, we describe the case of a woman who developed lung and mediastinal lymph node metastases two years after a previously cured triple-negative breast cancer. The patient initiated chemotherapy and, upon medical advice, decided to integrate it with O_2_/O_3_ therapy, MLT, and CBD. After 2 months, the lung nodules were no longer detectable, although lymphadenopathy persisted. Due to chemotherapy-related side effects, the patient discontinued systemic chemotherapy but continued the integrative therapies and underwent stereotactic radiotherapy. Three months later, the lymph node lesions had decreased in size. Subsequently, she started therapy with Olaparib while maintaining MLT, CBD, and O_2_/O_3_ therapy. Less than two years after the diagnosis of metastatic disease, the patient achieved a complete response.

In the following three years, she continued immunotherapy, interrupted for a few months due to nausea and leukopenia, alongside integrative therapy. Notably, she remains disease-free five years after the diagnosis of lung and mediastinal metastases and is still receiving Olaparib, MLT, CBD, and O_2_/O_3_ therapy.

The antiproliferative and chemosensitizing effects of MLT and CBD have been extensively demonstrated in vitro and in vivo, including in breast cancer models [7,9,10,11]. MLT has also been investigated in a clinical trial on breast cancer patients, although the results are still pending (NCT01965522). Clinical trials have tested cannabis-derived compounds, such as Nabiximols, in combination with chemotherapy, showing promising anticancer effects, particularly in glioma models [8]. Similarly, ozone therapy has been reported to inhibit breast cancer cell growth and enhance chemotherapy efficacy in vitro [5,6]. More recently, the combined use of CBD, MLT, and O_2_/O_3_ therapy demonstrated synergistic anticancer activity in preclinical models of pancreatic ductal adenocarcinoma [12], while another case report described a positive outcome in a glioblastoma multiforme patient treated with this integrative approach [13,14].

The remarkable clinical outcome observed in this patient occurred within a multimodal therapeutic framework. While the patient underwent a robust conventional protocol—including carboplatin/gemcitabine, stereotactic radiotherapy, and Olaparib—the integration of MLT, CBD, and O_2_/O_3_ therapy may have contributed to her treatment efficacy.

Although the individual contribution of each integrative agent towards tumor efficacy cannot be isolated from the standard regimen, their combined use proved to be safe and may have acted as a significant augmentative factor. This case supports the utility of such an integrative approach as a safe, supportive strategy in the management of TNBC.

Taken together, this case highlights the potential role of MLT, CBD, and O_2_/O_3_ therapy as adjuvant strategies capable of enhancing the efficacy of conventional oncological treatments. Further studies are warranted to clarify the clinical benefit and mechanisms underlying this integrative approach.

## 4. Conclusions

This case illustrates a sustained complete response in metastatic triple-negative breast cancer achieved within a multimodal therapeutic strategy that combined standard oncological treatments with integrative therapies. While the efficacy of conventional chemotherapy, radiotherapy, and Olaparib remains central, the long-term disease-free survival and favorable tolerability observed suggest that the adjunctive use of MLT, CBD, and O_2_/O_3_ therapy may have provided supportive and potentially synergistic benefits. Although causal relationships cannot be established from a single case, these findings support further clinical investigation into integrative approaches as safe adjuvant strategies to enhance treatment outcomes in TNBC.

## Data Availability

The original data presented in this study are available on reasonable request from the corresponding author. The data are not publicly available due to privacy concerns.

## Abbreviations

The following abbreviations are used in this manuscript:

CBD	cannabidiol
ER	estrogen receptors
ER+	ER-positive
HER2	human epithelial growth factor receptor 2
HER2+	HER2-positive
MLT	melatonin
O_3_	ozone
O_2_/O_3_	oxygen–ozone
PET	positron emission tomography
PR	progesterone receptor
ROS	reactive oxygen species
TAC	computerized axial tomography
TNBC	triple-negative breast cancer
